# Alpha- to Beta-Cell Transdifferentiation in Neonatal Compared with Adult Mouse Pancreas in Response to a Modest Reduction in Beta-Cells Using Streptozotocin

**DOI:** 10.3390/ijms252011152

**Published:** 2024-10-17

**Authors:** Jiwon Hahm, Bavina Thirunavukarasu, Reva Gadoo, Juan Andres Fernandez Andrade, Tyler Dalton, Edith Arany, David J. Hill

**Affiliations:** 1 Department of Physiology and Pharmacology, Schulich School of Medicine and Dentistry, Western University, London, ON N6A 3K7, Canada; jhahm2@uwo.ca (J.H.); bthiruna@uwo.ca (B.T.); juanfernandez2509@gmail.com (J.A.F.A.); tdalton4@uwo.ca (T.D.); 2 Lawson Health Research Institute, St. Joseph’s Health Care, London, ON N6A 4V2, Canada; gadoor1@mcmaster.ca (R.G.); earany@uwo.ca (E.A.); 3 Faculty of Science, McMaster University, Hamilton, ON L8S 4L8, Canada; 4 Department of Medicine, Schulich School of Medicine and Dentistry, Western University, London, ON N6A 3K7, Canada

**Keywords:** pancreas, beta-cell, alpha-cell, transdifferentiation, bi-hormonal, mouse, diabetes, streptozotocin

## Abstract

Following the near-total depletion of pancreatic beta-cells with streptozotocin (STZ), a partial recovery of beta-cell mass (BCM) can occur, in part due to the alpha- to beta-cell transdifferentiation with an intermediary insulin/glucagon bi-hormonal cell phenotype. However, human type 2 diabetes typically involves only a partial reduction in BCM and it is not known if recovery after therapeutic intervention involves islet cell transdifferentiation, or how this varies with age. Here, we used transgenic mouse models to examine if islet cell transdifferentiation contributes to BCM recovery following only a partial depletion of BCM. Cell lineage tracing was employed using Glucagon-Cre/yellow fluorescent protein (YFP) transgenic mice treated with STZ (25 mg/kg—neonates; 70 mg/kg—adults) or vehicle alone on 3 consecutive days. Mice were euthanized 2–30 days later with a prior glucose tolerance test on day 30, and immunofluorescence histology performed on the pancreata. Beta-cell abundance was reduced by 30–40% two days post STZ in both neonates and adults, and subsequently partially recovered in adult but not neonatal mice. Glucose tolerance recovered in adult females, but not in males or neonates. Bi-hormonal cell abundance increased 2–3-fold in STZ-treated mice vs. controls in both neonates and adults, as did transdifferentiated cells expressing insulin and the YFP lineage tag, but not glucagon. Transdifferentiated cell presence was an order of magnitude lower than that of bi-hormonal cells. We conclude that alpha- to beta-cell transdifferentiation occurs in mice following only a moderate depletion in BCM, and that this was accompanied by a partial recovery of BCM in adults.

## 1. Introduction

Islet cell replacement through the transplantation of cadaver- or human stem cell-generated human islets can reverse type 1 diabetes but is limited in its application through costs and tissue availability [1]. An alternative strategy for cell-based insulin replacement that could equally be applied to type 2 diabetes is beta-cell regeneration. An ideal candidate source would be proliferation from the remaining beta-cells. However, studies in rodent models of diabetes found that regeneration is limited and that new beta-cells mostly originated from the proliferation of pre-existing beta-cells, rather than from the differentiation of resident pancreatic progenitor cells [2,3]. Beta-cell proliferation significantly diminishes following childhood to around 0.2% of cells in humans and 1% in rodents during adulthood explaining why a contribution to the reversal of diabetes is minimal [4,5,6,7]. Similarly, the regeneration of beta-cell mass (BCM) from resident islet beta-cell progenitors or pancreatic ductal cells following experimental diabetes has been demonstrated but declines substantially with age [8,9,10,11]. Acinar cell to beta-cell transdifferentiation occurs only after extreme pancreatic damage, and can be reproduced in vitro by the transfection of key transcription factors defining the beta-cell phenotype such as *Pdx1*, *Ngn3*, and *MafA* [12,13]. In contrast, transdifferentiation from non-beta endocrine cells to generate new, functional beta-cells without the need for cell proliferation has been demonstrated for both alpha- and delta-cells, but with different age characteristics in rodents; delta- to beta-cell transdifferentiation being dominant in neonatal mice but alpha- to beta-cell transition being more frequent in adult animals [14]. However, these findings were observed only after near-total ablation of the beta-cells, and do not reflect the pathology of human type 2 diabetes where, even after protracted disease, the reduction in BCM is around 30–60% [15], largely due to apoptosis caused by post-transcriptional modifications to p53 as a result of increased reactive oxygen species within the beta-cells [16].

Alpha-cells constitute the largest population of endocrine cells other than beta-cells in both mouse and human islets [17] and represent a potential for therapeutic transdifferentiation in the expansion of BCM. Cells undergoing transdifferentiation typically have a common progenitor upstream of their development [18]. As such, bi-hormonal cells containing both glucagon and insulin exist throughout life in both mice and humans and may be intermediates in the transdifferentiation of alpha- to beta-cells [19]. Ahlgren et al. [20] utilized the disruption of *Pdx1* to cause a loss of mature beta-cell identity, which resulted in alpha-cell hyperplasia and an increased abundance of glucagon/insulin bi-hormonal cells in both the mantle and core of the islet. Similarly, alpha- to beta-cell transdifferentiation has been experimentally manipulated through the expression of transcription factors. For example, ectopic expression of *Pax4*, a defining transcription factor for beta-cells, induced the conversion of alpha-cells into insulin-producing cells [21]. A similar outcome was seen following the GABA-mediated downregulation of *Arx*, a transcription factor promoting alpha-cell fate [22]. Induced beta-cell injury without genetic manipulation also enhanced alpha- to beta-cell transdifferentiation as in the near total ablation of beta-cells in adult mice with targeted diphtheria toxin [23]. In contrast, metabolic insult alone, such as a low protein diet in utero to restrict beta-cell function postnatally, failed to induce a significantly higher level of such transdifferentiation compared to control conditions [24]. These studies suggest that a BCM restorative transdifferentiation process may be stimulus-dependent. In this study, we investigated the extent to which alpha- to beta-cell transdifferentiation could occur following a more limited depletion of BCM of 30–40% with moderate hyperglycemia, more closely representing that seen in human type 2 diabetes, using the beta-cell toxin, streptozotocin (STZ). The rationale was to identify if islet cell transdifferentiation could represent a potential therapeutic pathway for the reversal of type 2 diabetes.

Cell lineage fate tracing was used to identify alpha- to beta-cell transdifferentiation using a proglucagon promoter-Cre (Gcg-Cre) transgenic mouse strain which was crossed with mice expressing a yellow fluorescent protein (YFP) gene. Islet alpha-cells expressing proglucagon co-expressed Cre recombinase, which deleted loxP sites flanking a stop codon upstream of the YFP gene to allow YFP expression. Alpha-cells that transdifferentiated into islet cells expressing other hormone phenotypes continue to express YFP even in the absence of proglucagon gene expression. We also examined the effect of age on islet cell transdifferentiation by comparing young neonatal mice with adult animals. For adult mice, we used a tamoxifen-inducible Gcg-Cre transgenic mouse to avoid any influence of alpha- to beta-cell transdifferentiation earlier in life. The use of the inducible gene construct in neonatal mice was not possible given the need for a wash-out period after tamoxifen treatment.

## 2. Results

**Figure 1:**
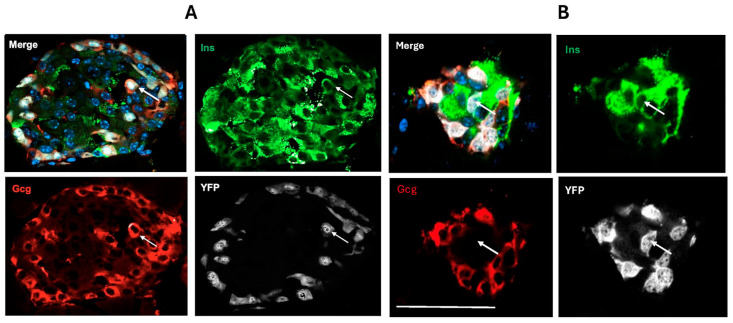
Immunohistochemical visualization of bi-hormonal cells (arrows) co-staining for insulin (Ins, green), glucagon (Gcg, red), and YFP (white) (**A**); and transdifferentiated cells (arrows) co-staining for insulin and YFP but not glucagon (**B**) within adult mouse pancreas. Cell nuclei are visualized with DAPI in the merged image. The size bar represents 100 μm in (**A**) and 50 μm in (**B**).

**Figure 2:**
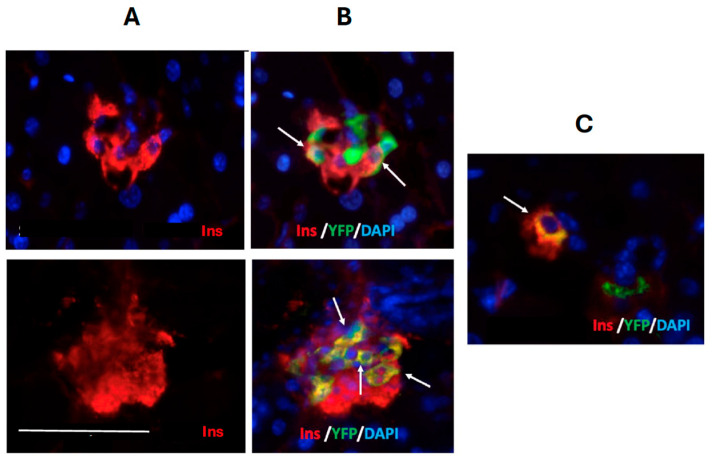
Immunohistochemical visualization of alpha- to beta-cell transdifferentiation in small islets (**A**,**B**) and an endocrine cell cluster (**C**) within adult mouse pancreas. Insulin (Ins) presence is shown in red and YFP presence in green. Cell nuclei are visualized with DAPI. Arrows indicate examples of alpha- to beta-cell transdifferentiation. The size bar represents 50 μm.

Both the Gcg-Cre/YFP construct used to investigate cell transdifferentiation in the neonatal mouse pancreas and the Gcg-CreERT/YFP used for adult mouse studies demonstrated a high efficiency of expression of YFP with greater than 85% of islet cells immunostaining for glucagon also expressing YFP. Bi-hormonal cells co-staining for insulin and glucagon in addition to YFP (Gluc^+^Ins^+^YFP^+^) were observed in the mantle and also within the core of islets at both ages, as were alpha- to beta-transdifferentiated cells (Gluc^−^Ins^+^YFP^+^) (Figure 1). The transdifferentiated cells were also seen in small islet cell clusters with greater abundance than in the larger islet structures (Figure 2).

### 2.1. Neonatal Mice

**Figure 3:**
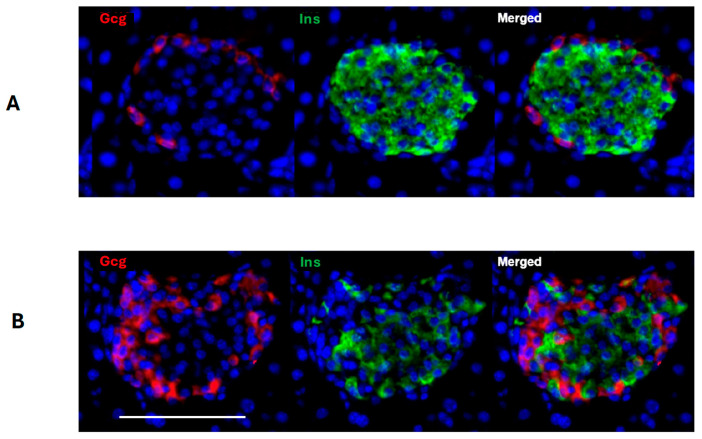
Immunohistochemical visualization of glucagon (Gcg, red) and insulin (Ins, green) in neonatal mouse islets two days following treatment with vehicle alone (**A**) or STZ (**B**). Cell nuclei are visualized with DAPI. A 31% mean reduction in insulin-containing beta-cells occurred following STZ treatment with an increased number of glucagon-containing alpha-cells extending into the core of the islet in addition to the rim. The size bar represents 100 μm.

Two days following the final injection of STZ, the targeted reduction in the proportion of beta-cells was approximately achieved (30.7%) when compared to the vehicle-treated animals. In addition, the cellular composition of the islet also differed between the control and STZ-treated animals, such that more glucagon immunoreactive cells were seen after STZ and were not limited to the islet mantle, as seen in controls, but were extended within the core of the islets (Figure 3).

**Figure 4:**
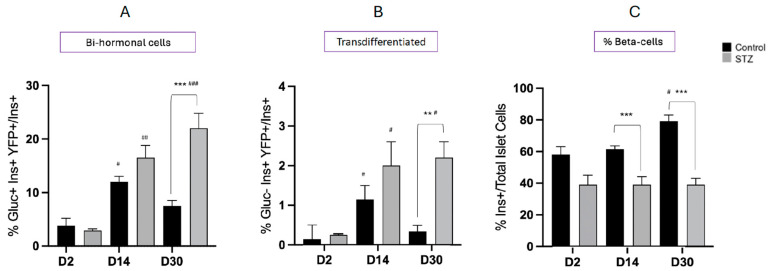
Changes in the abundance of glucagon/insulin bi-hormonal cells (**A**), alpha- to beta-transdifferentiated cells (**B**), and the percent insulin containing beta-cells/islet (**C**) in neonatal mouse pancreata 2 days (D2), 14 days (D14), or 30 days (D30) following treatment with STZ or vehicle alone (Control). Bi-hormonal cells were recognized as co-staining for glucagon (Gluc^+^), insulin (Ins^+^), and YFP (YFP^+^) and are expressed relative to total Ins^+^ cells. Transdifferentiated cells were recognized as co-staining for insulin and YFP, but not glucagon. Values show mean ± SEM. ^#^ *p* < 0.05, ^##^ *p* < 0.01, ^###^ *p* < 0.001 vs. D2; ** *p* < 0.01, *** *p* < 0.001 vs. control.

In the control mice, the relative abundance of bi-hormonal (Gluc^+^Ins^+^YFP^+^) cells was less than 5% of the insulin-immunoreactive islet cell population at study day 2 post-vehicle treatment, but significantly increased to over 10% at day 14. It then decreased by day 30, reflecting developmental changes in the bi-hormonal cell presence during neonatal life (Figure 4A). The presence of transdifferentiated cells (Gluc^−^Ins^+^YFP^+^) was an order of magnitude lower than that of bi-hormonal cells on day 2 but significantly increased at day 14 before decreasing by day 30 in a similar pattern to bi-hormonal cells (Figure 4B). In the control neonatal mice, there was no significant change in the percent contribution of insulin-positive cells/islet over the study period which represents 7 to 35 days following birth (Figure 4C). In mice receiving STZ, the abundance of bi-hormonal cells was 4-fold higher at day 30 than at day 2 and significantly greater than in control mice. Similar increases were found in the abundance of transdifferentiated cells. However, there was no recovery in the percentage of insulin-immunoreactive cells per islet after exposure to STZ. When male and female animals were examined separately, there were no significant differences in mean values, and the data are therefore combined in Figure 4.

**Figure 5:**
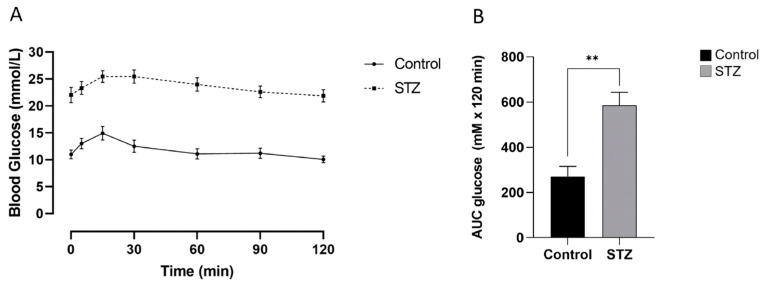
(**A**) Glucose excursions during intraperitoneal glucose tolerance tests in neonatal mice at D30 following treatment with STZ or vehicle alone (control); and (**B**) the glucose area under the curve (AUC). Values show mean ± SEM. ** *p* < 0.01 vs. Control.

We examined how glucose tolerance was altered in neonatal mice 30 days after STZ or vehicle alone by performing an IPGTT. Fasting blood glucose levels were still elevated in the animals that had received STZ compared to controls and the area under the curve (AUC) over the course of the GTT was significantly higher (Figure 5). No differences were observed between male or female animals.

### 2.2. Adult Mice

Similar studies were undertaken with adult mice using animals bearing the inducible Gcg-CreERT/YFP construct. At 2 days post-treatment with 70 mg/kg/day STZ, the mean reduction in the percentage of insulin immunoreactive cells/islet was 37.7%, similar to that achieved in neonatal mice but with nearly three times the dose of STZ. As in neonates, the cellular distribution within the islet differed between the control and STZ-treated animals, such that more glucagon immunoreactive cells were seen within the core of the islet 30 days following STZ compared to control mice.

**Figure 6:**
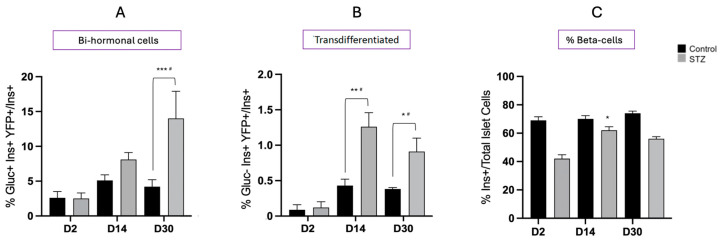
Changes in the abundance of glucagon/insulin bi-hormonal cells (**A**), alpha- to beta-transdifferentiated cells (**B**), and the percent insulin containing beta-cells/islet (**C**) in adult mouse pancreata 2 days (D2), 14 days (D14), or 30 days (D30) following treatment with STZ or vehicle alone (control). Bi-hormonal cells were recognized as co-staining for glucagon (Gluc^+^), insulin (Ins^+^), and YFP (YFP^+^) and are expressed relative to total Ins^+^ cells. Transdifferentiated cells were recognized as co-staining for insulin and YFP, but not glucagon. Values show mean ± SEM. ^#^ *p* < 0.05 vs. D2; * *p* < 0.05, ** *p* < 0.01, *** *p* < 0.001 vs. control.

In control animals, the relative abundance of bi-hormonal cells was 2.6% of the total insulin-immunoreactive cell population at day 2 and did not significantly change at days 14 or 30 (Figure 6A). As with neonates, the abundance of transdifferentiated cells was an order of magnitude lower than that of bi-hormonal cells on day 2 and did not change at days 14 and 30 (Figure 6B). Similarly, no change in the percentage abundance of beta-cells/islet occurred in control mice between study days 2 and 30 (Figure 6C). In mice receiving STZ, the abundance of bi-hormonal cells increased 6-fold between days 2 and 30 and was significantly higher than in controls at day 30 (Figure 6A), as was the presence of transdifferentiated islet cells at days 14 and 30 (Figure 6B). The percentage of insulin-immunoreactive cells per islet significantly increased between days 2 and 14 following STZ but did not improve further at day 30 (Figure 6C). Hence, a partial recovery of the beta-cell population had occurred after STZ in adult mice and was accompanied by an increased abundance of bi-hormonal and transdifferentiated cells. No differences were found between males and females, and combined data are shown.

**Figure 7:**
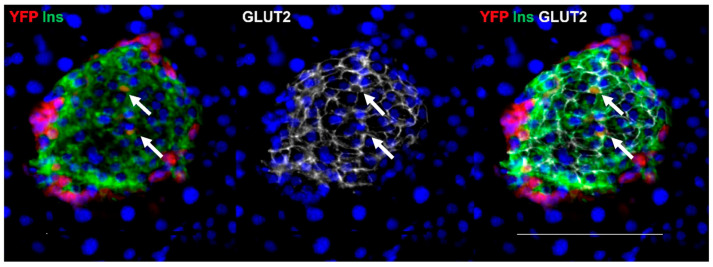
Immunohistochemical visualization of insulin (Ins, green), YFP (red), and Glut2 (white) in an adult mouse islet. Cell nuclei are visualized with DAPI. Alpha- to beta-transdifferentiated cells shown by co-localization of Ins and YFP also express Glut2 (arrows). The size bar represents 100 μm.

To assess the likelihood that the increased abundance of alpha- to beta- transdifferentiated cells seen following STZ treatment in adult mice represented functional beta-cells, we examined the presence of Glut2 in cells staining positive for YFP and insulin on day 30 after STZ. Glut2 was present on the cell membranes of most insulin immunoreactive cells in the core of the islets, including many transdifferentiated cells (Figure 7).

**Figure 8:**
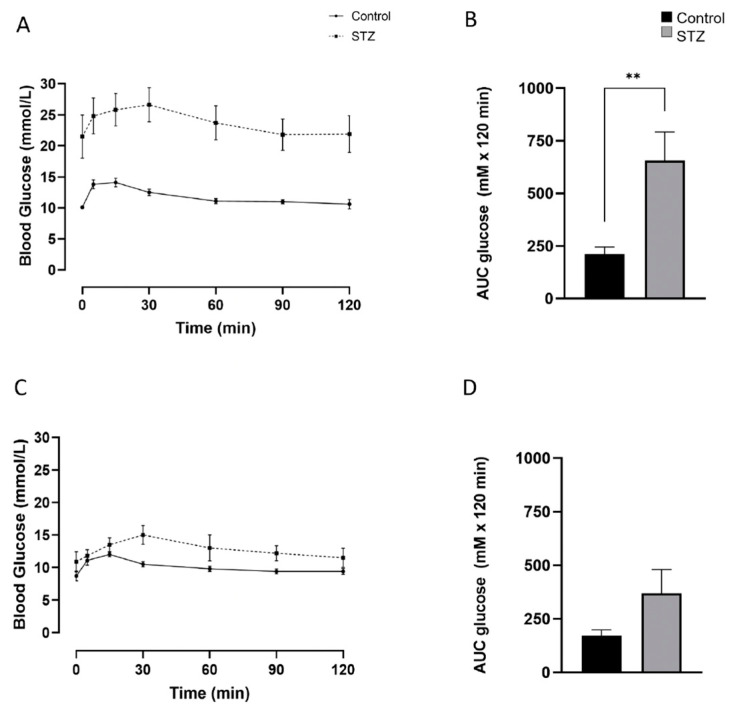
Glucose excursions during intraperitoneal glucose tolerance tests and area under the curve (AUC) in adult male (**A**,**B**) and female (**C**,**D**) mice at D30 following treatment with STZ or vehicle alone (control). Values show mean ± SEM. ** *p* < 0.01 vs. control.

Glucose tolerance tests were performed in adult mice 30 days after receiving STZ or vehicle. Fasting blood glucose levels tended to be higher in males than in females that had received STZ compared to controls. The AUC remained significantly higher after STZ compared to controls for male mice, but not so in females although the mean value trended higher in females after STZ treatment (Figure 8).

### 2.3. Comparison Between Neonatal and Adult Mice

**Figure 9:**
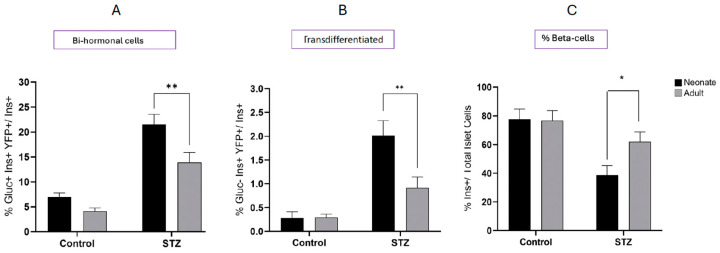
Comparison of glucagon/insulin bi-hormonal (**A**), transdifferentiated cell presence (**B**), and the percent insulin containing beta-cells/islet (**C**) between neonatal and adult mouse pancreata 30 days following treatment with STZ or vehicle alone (control). Values show mean ± SEM. * *p* < 0.05, ** *p* < 0.01 vs. neonates.

We compared the neonatal and adult mice with respect to the relative abundance of bi-hormonal cells and transdifferentiated cells on day 30 following STZ treatment or vehicle alone (Figure 9). The increase in bi-hormonal cells following STZ was significantly greater in neonatal compared to adult mice, as was the abundance of alpha- to beta-transdifferentiated cells. However, the recovery of the beta-cell population was only seen in the adult mouse pancreas.

## 3. Discussion

We investigated the extent to which alpha- to beta-cell transdifferentiation occurred in neonatal and adult mice as an adaptive mechanism to restore beta-cell abundance following a moderate reduction in BCM of the magnitude associated with human type 2 diabetes. The concentration of STZ needed to reduce the population of insulin-immunoreactive beta-cells by approximately 30–40% was found to be higher in adult mice at 70 mg/kg BW compared to the 25 mg/kg required in neonates, each administered over three days. Previous studies reported that two daily injections of 40 mg/kg STZ in adult mice of the same background as used here reduced the mean islet area by 35% but without quantification of the specific loss of beta-cells [25]. When GcgERT/YFP mice, as used in the present study, were administered 50 mg/kg STZ over five days at 10 weeks of age the relative reduction in mean beta-cell area/islet was 30% [26]. The heightened response to STZ in neonatal mice found here contradicts previous studies in rats where sensitivity was shown to increase with age [27,28]. Since the beta-cell phenotype in neonatal rodents is immature, characterized by lower GSIS than in adults with a relatively lower expression of GLUT2 [29] by which STZ is able to be transported into beta-cells, it might be anticipated that a greater concentration of STZ would be required in the neonate. The reverse finding may be linked to the use of a tamoxifen-inducible construct here for the adult animals, although the tamoxifen wash-out period was similar to that utilized by others [26]. A more likely explanation relates to the ontological changes in beta-cell phenotype occurring in early postnatal life. Previously it was shown that rat islets undergo structural remodeling between birth and weaning involving a loss of the immature beta-cell population through apoptosis, beginning at 6 days and peaking at 2 weeks of age, with a replacement by more mature beta-cells with acute glucose-sensitivity [30,31]. It is possible that this new cohort of beta-cells was destroyed by STZ in the neonate before being able to replicate and replete a GSIS-competent beta-cell population. This would be supported by the observation here that even modest STZ treatment of neonatal mice resulted in persistent hyperglycemia after 30 days with a poor recovery of beta-cell abundance. While long-term hyperglycemia can limit beta-cell regeneration after experimental loss [32], this cannot account fully for the failure of beta-cell abundance to recover in neonates since this significantly recovered in adult mice with an equivalent fasting glucose value, at least in males.

Maturity of the Unfolding Protein response (UPR) may also contribute to the inability of BCM to recover following moderate loss of BCM in the neonatal mice compared to adults. The increased demand for insulin secretion from remaining beta-cells can result in endoplasmic reticular (ER) stress, depleting the ER of Ca^2+^ stores, and activating the UPR to limit protein translation and reduce protein misfolding [33]. While in the short-term this a beneficial adaptive response to limit beta-cell stress, if ER stress is prolonged the UPR response can lead to increased protein misfolding and activate beta-cell apoptosis [34]. Insulin-expressing beta-cells generated in vitro from human pluripotential stem cells are initially immature with poor GSIS, a phenotype similar to that present in the neonatal mouse pancreas, but with high expression of transcription factors such as XBP1 which can activate the transcription of genes leading to an ER stress response [35], resulting in apoptosis. Thus, the generation of beta-cell ER stress in the neonatal mouse following beta-cell depletion may amplify the developmental apoptosis normally present at this age and thereby limiting the recovery of BCM.

In the adult mice, glucose tolerance observed 30 days following STZ varied by sex. Females, but not males, treated with STZ recovered glucose tolerance comparable to controls, except for the delayed timing of the blood glucose peak. The greater functional recovery in females compared to males following STZ administration has been reported previously [36,37]. Tanday et al. [38] compared glycemia and islet beta-cell area in adult GcgERT/YFP mice 11 days after STZ treatment. While glucose tolerance did not differ between the sexes at this early timepoint there was a greater retention of beta-cells in females with less evidence of ongoing beta-cell apoptosis than in males. Conversely, the frequency of glucagon/insulin bi-hormonal cells was higher in males which could represent a greater adaptive transdifferentiational response within the alpha-cell population. The greater resilience of beta-cells in females to STZ-induced apoptosis may be due to a protective effect of estrogens on survival and function, potentially mediated through estrogen receptor-alpha signaling [39,40]. However, serum estrogen levels in female C57BL mice are negligible until approximately 26 days of age [41], which may explain the absence of any observed sex differences in glucose tolerance following STZ treatment in the neonatal group.

Glucagon/insulin bi-hormonal cells within islets represented approximately 3–4% of insulin-immunoreactive cells in neonates and 2.5% in adult mice. Their abundance increased to approximately 15–20% of insulin-immunoreactive cells 30 days following STZ treatment being significantly greater in neonates than in adults. All of the observed bi-hormonal cells contained the alpha-cell lineage YFP tag and hormone-null YFP-expressing cells were rarely seen, suggesting that there was little dedifferentiation of alpha-cells to a hormone-null state prior to transdifferentiation into beta-cells. The presence of insulin/glucagon bi-hormonal cells in the adult GcgERT/YFP mouse pancreas has been reported previously [42], but our results in neonatal mice differ from those of Chera et al. [14] who noted that endocrine cell transdifferentiation to yield new beta-cells occurred exclusively from somatostatin-expressing delta-cells prior to six weeks of age. The presence of bi-hormonal cells in the pancreata of control neonatal mice may be a normal facet of islet development since a significant increase in their abundance was seen between 7 (experimental day 2) and 19 days of age, before a subsequent decline at 35 days. This suggests that an increase in glucagon/insulin bi-hormonal cells are part of the anatomical and functional islet restructuring that happens between birth and weaning.

Human studies also support a role for bi-hormonal cells during islet development. When beta- and alpha-cell development was modeled in vitro from human embryonic stem cells, a glucagon/insulin bi-hormonal cell population was observed at the stage of endocrine cell maturation [43]. Similarly, insulin/glucagon bi-hormonal cells have been observed in the developing human pancreas but were much reduced in the adult pancreas [44,45]. Such cells have a transcriptomic signature that is most similar to mature alpha-cells and have been shown to differentiate into monohormonal alpha-cells [46], suggesting that bi-hormonal cells do not always indicate alpha- to beta-cell transdifferentiation. An increased abundance of bi-hormonal cells was reported within the islets of lean patients with type 2 diabetes [47], and in non-diabetic individuals with poor insulin sensitivity [48], suggesting that this may represent a compensatory response involving islet cell transdifferentiation. However, it could equally indicate a dedifferentiation of beta-cells under metabolic stress, as has been reported to occur in type 2 diabetes [49].

Transdifferentiation of alpha-cells to insulin monohormonal cells was found to be a rare event in neonatal mice at 7 days of age with a frequency of around 0.2% of all insulin-staining cells. However, the abundance increased 10-fold by 19 days of age before declining by 35 days. This ontology mirrored the changes in bi-hormonal cells and suggests that around 10% of bi-hormonal cells were able to fully differentiate into insulin monohormonal cells during neonatal islet development. Following STZ treatment of neonatal mice, no change in transdifferentiated insulin monohormonal-cell abundance at days 2 or 14 days after STZ was found, but such cells were significantly increased by 30 days as assessed by the co-localization of YFP with insulin. Transdifferentiation was also seen with a higher frequency in small islets and in extra-islet endocrine clusters. Such cell clusters are abundant in both mouse and human pancreata throughout life, and we have previously observed them to be enriched in immature beta-cell progenitors with high proliferative activity (11). The relative abundance of transdifferentiated beta-cells was lower in adults than in neonates but similarly increased following partial reduction in BCM with STZ. Previously, the appearance of new beta-cells by transdifferentiation has only been reported after the near-total removal of beta-cells, as seen following the administration of diphtheria toxin (DT) to transgenic mice specifically expressing the diphtheria toxin receptor within beta-cells [23], or following a more gradual loss of beta-cells using the ‘pancreatic islet beta-cell apoptosis through targeted activation of caspase 8′ mouse model (PANIC-ATTAC) [50,51]. In these reports, up to a 75% loss of beta-cells could be reversed over 10 weeks with a major contribution coming from alpha- to beta-cell transdifferentiation. In studies of human pancreata from patients with type 1 diabetes, the frequency of insulin/glucagon bihormonal islet cells was found to be higher than in non-diabetics [52], suggesting that a similar regenerative pathway might also exist.

The generation of new beta-cells from alpha-cells under moderate glycemic stress should be considered in the context of other adaptations that occur within the islets of Langerhans. A reduction in BCM of approximately 50% was achieved previously using the DT method without the appearance of overt hyperglycemia [53]. A transient increase in the proliferation of remaining beta-cells was found, associated with an increase in the expression of both insulin and glucagon. An increase in proglucagon gene expression was observed in other models of beta-cell regeneration associated with an expansion in alpha-cell numbers [25]. Alpha-cell presence and glucagon gene expression and action are obligatory for beta-cell regeneration [54] and have been linked with an expression of prohormone convertase 1/3 by alpha cells resulting in an increased processing and synthesis of GLP-1 within the damaged islets [55]. Paracrine GLP-1 not only acts as a mitogen for the remaining beta-cells [56] but has been identified as a trigger for alpha- to beta-cell transdifferentiation [57]. The present study expands the previous reports to show that as little as a 30–40% reduction in beta-cell abundance using STZ can trigger alpha- to beta-cell transdifferentiation within a 30-day window in both neonatal and adult mice. However, given the low incidence of transdifferentiated cells, the recovery of beta-cell mass in adult mice is likely to have also involved other mechanisms such as the proliferation of remaining beta-cells, the generation of beta-cells by neogenesis from resident beta-cell progenitors as previously observed by us [10], or by transdifferentiation from non-alpha-cell types such as delta-cells.

The lower proportion of transdifferentiated cells observed here in the damaged adult pancreas compared with neonates may indicate that this mechanism of cellular plasticity decreases with age but is not completely lost. This suggests a therapeutic potential for enhancing the generation of bi-hormonal cells and alpha- to beta-cell transdifferentiation by the administration of candidate exogenous agents, such as GLP-1. In addition to the administration of GLP-1 receptor agonists [58], the paracrine presence of endogenous GLP-1 might be enhanced by the administration of dipeptidyl peptidase 4 (DPP-4) to prevent the degradation of GLP-1, which has been shown to potentiate alpha- to beta-cell transdifferentiation in the diabetic mouse [59]. Antagonism of the glucagon receptor has also been shown to upregulate islet GLP-1 production [60]. Other potential agents include GABA [22,61], taurine supplementation to diet [26], and artemisinins [62]. The findings around the artemisinin action on islet cell transdifferentiation have, however, lacked consistency [63]. Future research should examine the actions of these or other candidate molecules in enhancing BCM through islet cell transdifferentiation in a variety of animal models of type 2 diabetes.

A technical limitation of this study was the reliance on immunohistochemical analyses for the identification of transdifferentiating cells. While immunohistochemistry allowed us to detect the co-localization of YFP and insulin, it did not provide insights on the functional ability of transdifferentiated cells to secrete insulin following a glucose stimulus. However, in many bi-hormonal and transdifferentiated cells, we detected the presence of Glut2, representing at least one biomarker of glucose sensitivity and potential functionality. This would be consistent with a transcriptional signature of transdifferentiated cells measured previously, included the expression of key transcription factors such as *Pdx1*, *Ngn3*, and *Nkx6.1* [64]; all necessary for glucose-dependent insulin synthesis and release. Future studies might separate YFP-expressing insulin monohormonal cells from disaggregated islets using fluorescence-activated cell sorting to examine their capacity for glucose-stimulated insulin secretion, although the numbers of such cells within an individual pancreas are small. Additionally, a longer follow-up of the fate of transdifferentiated cells beyond 30 days post-STZ is also advisable in subsequent studies to determine the longevity of such cells. Since beta-cell abundance recovered poorly in neonatal mice following STZ, a longer follow-up period may show a greater degree of regeneration, although the studies of Cox et al. [65] extending to 130 days in mice suggested that a full recovery of beta-cell mass did not occur and glucose intolerance was seen at the older ages. Also, because the mice were still hyperglycemic at 30 days, it is possible that this was having a negative effect on regenerative capacity, which could be corrected by insulin therapy.

Prior to the consideration of therapeutic implications, it must also be recognized that there exist inherent differences between the anatomy of rodent and human islets [66]. In particular, humans and rodents exhibit a differing juxtaposition of alpha-cells and beta-cells, with alpha-cells being concentrated in the mantle within mouse islets but distributed throughout the islets in humans [67]. This may have important ramifications for the paracrine interactions between the two cell types, particularly for the locally acting molecules such as GLP-1 or GABA that contribute alpha- to beta-cell transdifferentiation. These studies further highlight the potential role of manipulation of alpha-cells in the regeneration of beta-cell mass following even a partial loss of beta-cell, as occurs in human type 2 diabetes.

## 4. Materials and Methods

### 4.1. Animal Models

Procedures involving animals were performed in accordance with the animal use protocol approved by the Animal Care Committee at Western University and consistent with the guidelines of the Canadian Council for Animal Care (Approval numbers AUP 2018–027 and AUP 2022–070). Mice were given water and standard rodent diet ad libitum and were maintained in a temperature-controlled room with a 12 h light/dark cycle at the Lawson Health Research Institute, London, Ontario, Canada.

To trace the lineage of alpha-cells in neonatal mice, we used a proglucagon promoter-Cre (Gcg-Cre) transgenic mouse strain (The Jackson Laboratory, Bar Harbor, ME, USA; Catalogue #030663) [68]. Gcg-Cre mice were generated from a C57BL/6 background with a Cre-recombinase downstream of the proglucagon gene promoter and were maintained as a homozygous strain. The Gcg-Cre mice were crossed with Rosa26-enhanced yellow fluorescent protein (YFP) mice (The Jackson Laboratory; Catalogue #006148) [69]. Starting on postnatal day 2, mice were administered intraperitoneal injections of 25 mg/kg body weight (BW) STZ in 0.1M sodium citrate buffer, or an equal volume of sodium citrate buffer vehicle, for 3 consecutive days to eliminate approximately 30–40% of the beta-cell population. Animals were sacrificed by CO_2_ asphyxia at days 2, 14, and 30 following the final treatment with STZ and the pancreata were collected and processed for histology. We were unable to utilize an inducible model of Cre-recombinase in neonatal mice due to insufficient time being available for tamoxifen wash-out following birth before treatment with STZ.

For lineage tracing of alpha-cell fate in adult mice, we utilized animals containing an inducible proglucagon-CreERT2 construct (The Jackson Laboratory; Catalogue #030681) [70]. The rationale to use the inducible model was to examine the lineage fate of alpha-cells after the pancreas was functionally mature at 6–8 weeks of age without the influence of any transdifferentiation events that had occurred in fetal or neonatal development. Proglucagon-CreERT2 mice were crossed with C57BL/6 wild-type mice (The Jackson Laboratory; Catalogue #000664) to maintain a heterogeneous lineage. The litters were genotyped via polymerase chain reaction and heterozygotes were selected to be crossed with Rosa26-eYFP mice to produce a Gcg-CreERT2/Rosa26-eYFP (Gcg-CreERT/YFP) inducible double transgenic mouse strain. Adult mice were treated with tamoxifen (Sigma-Aldrich, St. Louis, MO, USA) by intraperitoneal injection in 30 mg/mL corn oil solution at 100 µg/g BW for 3 consecutive days [71]. Following tamoxifen injection, a washout period of 5 days occurred before treatment with STZ by intraperitoneal injections (70 mg/kg BW in 0.1M sodium citrate buffer) or an equal volume of vehicle alone, each administered for 3 consecutive days. The dose was titrated to achieve a similar level of beta-cell depletion at around 30–40% as seen in the non-inducible neonatal mouse model. Mice were euthanized by CO_2_ asphyxiation as above at 2, 14, and 30 days following the last injection with STZ and the pancreata were collected and processed for histology. Between 4 and 6 individual male and female mice were studied at each time point for both neonatal and adult mice.

### 4.2. Glucose Tolerance Test

Prior to euthanasia of mice on day 30, an intraperitoneal glucose tolerance test (IPGTT) was performed. Mice were fasted for 6 h prior to the injection of 2 g/kg glucose solution and approximately 1 µL of blood subsequently collected from the tail vein. Blood glucose levels were measured at 5, 15, 30, 60, 90, and 120 min following glucose injection using a OneTouch blood glucose monitor (LifeScan, Malvern, PA, USA). For each IPGTT curve, an area under the curve (AUC) was calculated with the exclusion of the area below the initial fasting blood glucose level to quantify the displacement of blood glucose from the fasting level [72].

### 4.3. Immunohistochemistry

Pancreata collected from neonatal and adult mice were fixed in 4% (*v*/*v*) paraformaldehyde for 24 h at 4 °C and rinsed in PBS. The fixed tissues were dehydrated in 30% (*v*/*v*) sucrose overnight prior to embedding in Tissue Plus optimal cutting temperature compound (OCT) (Thermo Fisher Scientific, Waltham, MA, USA), then frozen at −80 °C for further analysis. The frozen tissues were then sectioned with a Leica CM 1850 Cryostat at 7 µM thickness, with three replicate sections from each pancreas being collected with >100 µM intervals between each section. The interval was determined by the mean islet size of 6-week-old mice reported in the literature as s = 7.96 ± 0.57, where s = 1 was an average single cell diameter. Given that an average mouse beta-cell has a diameter between 13 and 18 µM, a minimum of 103.48 µM, or approximately 100 µM, was determined to be an appropriate interval to avoid a cellular overlap in the representation of an islet at different sections [73,74]. Slides were mounted on Superfrost Plus Micro Slides (VWR International, Radnor, PA, USA).

Heat-induced epitope retrieval (HIER) was performed in a pressurized chamber (decloaker) at 100 °C for 15 min in sodium citrate buffer (10 mM sodium citrate, 0.05% Tween 20, pH 6.0). Sniper universal block (Biocare Medical, Concord, CA, USA) was applied to tissues and incubated for 8 min to minimize non-specific binding. A mixture of glucagon (Mouse anti-glucagon 1:2000; Sigma-Aldrich, St. Louis, MO, USA); insulin (Guinea pig anti-insulin 1:20, Thermo Fisher Scientific); and YFP (Rabbit anti-GFP 1:200, Novus Biologicals, Englewood, CO, USA) primary antibodies were applied to slides and incubated at 4 °C overnight in a humidified chamber. The following day, specific secondary fluorescent antibodies were applied (Goat anti-guinea pig, Alexa Fluor 647, Goat anti-mouse, Alexa Fluor 555 or Goat anti-rabbit, Alexa Fluor 488, all at 1:500 dilution; Thermo Fisher Scientific) for 90 min and 4′,6-diamidino-2-phenylindole dihydrochloride (DAPI) (Thermo Fisher Scientific) was used to counterstain the nuclei. Coverslips were mounted onto the slides using a fluorescence mounting medium (Dako Canada, Burlington, ON, Canada) and sealed with a clear nail polish. In order to further characterize the functional maturity of transdifferentiated cell, we performed immunohistochemistry for Glut2 (Mouse anti-Glut2 1:100; Santa Cruz Biotechnology, Dallas, TX, USA).

### 4.4. Image Analysis

For this study, an islet of Langerhans was defined as a structure with greater than 6 endocrine cells staining for either insulin, glucagon, or both and surrounded by a basement membrane. Structures with less than 6 such cells were defined as an endocrine cell cluster. A Nikon Eclipse T2SR inverted microscope (Nikon, Minato, Tokyo, Japan) located at the Lawson Health Research Institute or a Nikon CSU-W1 Spinning Disk Confocal microscope located at the Biotron Facility, Western University were used to image tissue sections at 20× or 60×. Quantification of cell number was performed using Fiji ImageJ (https://www.nature.com/articles/nmeth.2089, accessed on 4 October 2024) [75] to quantify YFP^+^ cells that co-stained with glucagon alone (Gluc^+^Ins^−^YFP^+^), with both insulin and glucagon (Gluc^+^Ins^+^YFP^+^), or with insulin alone (YFP^+^Gluc^−^Ins^−^); the latter representing transdifferentiated endocrine cells. To quantify total Ins^+^, Gluc^+^, and YFP^+^ cells, an area analysis macro was used in Fiji ImageJ. The macro was developed by our laboratory to transform single channel images of insulin, glucagon, and YFP signals into binary images using the Phansalkar method to distinguish areas with or without a signal [76]. The area size with a positive signal was measured and corrected by the average area of a single alpha- or beta-cell to estimate the number of Ins^+^, Gluc^+^, or YFP^+^ cells in the imaged islet. Co-staining cells were identified and manually counted by assigning two antigens of interest with red and green filters where areas of overlap would display an orange color.

### 4.5. Statistical Analysis

Each animal was considered as a single observation and at least two sections were compared from the pancreas of each animal. Data were presented as means ± SEM with between three and six replicate animals being examined at each experimental time point for histological analyses, and seven to ten animals for glucose tolerance measurements. Data were analyzed for statistical differences between groups using one- or two-way ANOVA with Sidak’s multiple comparisons test to compare values within the same timepoint (GraphPad Prism Version 8, GraphPad Software, Boston, MA, USA).

## Data Availability

The authors will make available data generated within these studies upon request.
